# Impulse Control Disorders in Parkinson’s Disease: From Bench to Bedside

**DOI:** 10.3389/fnins.2021.654238

**Published:** 2021-03-12

**Authors:** Andrea Augustine, Catharine A. Winstanley, Vaishnav Krishnan

**Affiliations:** ^1^Department of BioSciences, Rice University, Houston, TX, United States; ^2^Department of Psychology, Djavad Mowafaghian Centre for Brain Health, The University of British Columbia, Vancouver, BC, Canada; ^3^Departments of Neurology, Neuroscience and Psychiatry & Behavioral Sciences, Baylor College of Medicine, Houston, TX, United States

**Keywords:** impulse control disorders, rodent models, dopamine agonist, non-motor symptoms, Parkinson’s disease

## Abstract

Parkinson’s disease (PD) is a neurodegenerative disorder that is characterized by symptoms that impact both motor and non-motor domains. Outside of motor impairments, PD patients are at risk for impulse control disorders (ICDs), which include excessively disabling impulsive and compulsive behaviors. ICD symptoms in PD (PD + ICD) can be broadly conceptualized as a synergistic interaction between dopamine agonist therapy and the many molecular and circuit-level changes intrinsic to PD. Aside from discontinuing dopamine agonist treatment, there remains a lack of consensus on how to best address ICD symptoms in PD. In this review, we explore recent advances in the molecular and neuroanatomical mechanisms underlying ICD symptoms in PD by summarizing a rapidly accumulating body of clinical and preclinical studies, with a special focus on the utility of rodent models in gaining new insights into the neurochemical basis of PD + ICD. We also discuss the relevance of these findings to the broader problem of impulsive and compulsive behaviors that impact a range of neuropsychiatric syndromes.

## Introduction

Parkinson’s disease (PD) affects more than 10 million people worldwide (Dorsey et al., 2018; Rocca, 2018). Motor symptoms of PD are best appreciated as a cardinal “triad” of tremor, rigidity, and bradykinesia and can include akinesia and postural instability in late stages (Jankovic, 2008). Additionally, a non-motor prodromal phase, which includes changes in sleep, mood, olfactory, and autonomic function, may precede motor symptoms by up to a decade (Chaudhuri et al., 2006; Ishihara and Brayne, 2006; Masala et al., 2018). Unfortunately, non-motor symptoms often persist during the motor phase of PD, compounding overall disability and augmenting the need for institutionalization. This review focuses on one particular constellation of non-motor symptoms termed “impulse control disorders” (ICDs) and highlights recent studies that provide important updates on pathophysiology.

The pathological hallmark of PD is depigmentation, cell loss, and gliosis within the substantia nigra (SN) (Dauer and Przedborski, 2003), which is comprised of dopaminergic neurons that project to the striatum to form the nigrostriatal dopamine pathway, crucial for the stimulation of voluntary movement (Haber, 2014). Lewy bodies, neurotoxic structures composed mainly of abnormally phosphorylated and aggregated alpha-synuclein, are thought to propagate nigral degeneration (Kouli et al., 2018) and subsequently extend beyond nigral circuits. In fact, the rostro-caudal extent of alpha-synuclein accumulation has been extensively explored as a potential etiology for non-motor symptoms that affect multiple neural systems, including rapid eye movement sleep behavior disorder (pontine and medullary nuclei), constipation (autonomic enteric neurons), and cognitive impairment (frontal and other cortical regions) (Adler and Beach, 2016; Atik et al., 2016).

Impulse control and related behaviors (ICRBs) are characterized by the inability to assert self-control in emotions and behaviors, which can lead to compulsive and/or impulsive actions that harm oneself or others (American Psychiatric Association, 2013). Compulsivity includes repetitive and consciously unwanted (ego-dystonic) behaviors that subjects describe as being necessary to perform (Berlin and Hollander, 2014). Impulsivity is the reduction or absence of forethought or planning before making decisions and can be divided into motor and decision impulsivity (Winstanley, 2011). Common ICDs experienced by PD patients include pathological gambling, binge eating, hypersexuality, and compulsive buying. ICD development in PD patients (PD + ICD) is commonly observed as an iatrogenic side effect of dopamine agonists (DAAs), a type of dopamine replacement therapy (DRT), and symptoms usually improve with reduction of DAA treatment (You et al., 2018; Kelly et al., 2020). In the following sections, we critically examine preclinical and clinical data that explore the neuroanatomical and molecular bases for this pathophysiological synergy. Importantly, we conclude by highlighting the significance of the rodent model in pushing this research niche forward and discussing broader implications for the more general problem of ICRBs that are a feature of several neuropsychiatric syndromes. Articles reviewed in this manuscript were identified through a PubMed search utilizing keyword combinations including “Parkinson’s disease,” “impulse control disorders,” “rat or mouse,” and “animal model.”

### Mechanisms Underlying PD + ICD: Human Studies

About 14–40% of PD patients experience symptoms of ICDs (Erga et al., 2017; Baig et al., 2019). Risk factors for PD + ICD include duration and dose of DAA treatment, male gender, comorbid apathy, anxiety, and depression (Weintraub et al., 2010a, 2013). DAA treatment, in particular, is strongly associated with a significantly increased risk of developing ICDs (Bódi et al., 2009; Garcia-Ruiz et al., 2014). Further, various psychological factors such as increased stress, greater illness identity, and negative coping strategies have also been found to predict PD + ICD (Garlovsky et al., 2016). ICD symptoms in newly diagnosed drug-naïve PD patients actually seem to be comparable to that of the general population, around 20% (Antonini et al., 2011; Weintraub et al., 2013). However, recent research has suggested a difference in the nature of ICRBs among these populations, whereby delay discounting (with preference for smaller and more immediate rewards over larger but more delayed rewards) and deficits in reflection impulsivity (ability to collect sufficient information prior to decision-making) better predict ICDs in the PD and general populations, respectively (Izzo et al., 2020).

Alterations in dopamine receptor signaling are a central property of PD + ICD. D1-like receptors (D1R), which include D1 and D5, are coupled to excitatory cyclic AMP (cAMP) signaling, and are broadly implicated in reward-based learning (Nakanishi et al., 2014). D2-like receptors (D2R), which include D2, D3, and D4, are coupled to cAMP inhibition and play a prominent role in avoidance learning (Draoui et al., 2020). DAAs such as pramipexole, ropinirole, rotigotine, and apomorphine are much more selective for D2R than D1R (Millan et al., 2002). Positron emission tomography (PET) approaches have indicated decreased D2R binding (Steeves et al., 2009; Stark et al., 2018) and relatively unchanged D1R binding in the ventral striatum in PD + ICD (Payer et al., 2015) compared with PD patients without ICDs (PD-ICD). These findings suggest that ICD symptoms may reflect DAA-mediated D2R overstimulation preventing punishment learning wherein adverse consequences of actions are ignored when making decisions, while the effects of D1R-mediated direct pathway and positive reinforcement learning are intact.

The density of dopamine transporters (DAT), responsible for the reuptake of dopamine from the synaptic cleft, may also play a key role. Single photon emission computed tomography (Cilia et al., 2010; Voon et al., 2014; Navalpotro-Gomez et al., 2019) and PET (Lee et al., 2014) investigations have demonstrated reduced striatal DAT binding in PD + ICD compared to PD-ICD. Furthermore, two studies have shown that decreased DAT availability precedes ICD development following DRTs and that the severity of ICDs experienced is inversely correlated with DAT density (Vriend et al., 2014; Smith et al., 2016), highlighting the potential use of baseline DAT density as a marker of future ICD risk in PD patients.

In studies of network-level dysfunction, resting-state functional magnetic resonance imaging (fMRI) approaches in medicated PD + ICD patients have indicated reduced cortico-striatal connectivity (Carriere et al., 2015; Ruitenberg et al., 2018) between the cingulate cortex and the nucleus accumbens (Hammes et al., 2019) and dorsal striatum (Cilia et al., 2011; Tessitore et al., 2017a). fMRI studies in which medicated subjects performed tasks to measure impulsivity have also observed lower activation in the prefrontal cortex (PFC) and striatum during these tasks in PD + ICD (Filip et al., 2018). Interestingly, several studies have identified more diffuse alterations in neural networks in newly diagnosed, drug-naïve PD patients who later developed ICDs or exhibited greater impulsivity compared to PD-ICD patients and healthy controls. This includes disruptions to numerous white matter tracts (Mojtahed Zadeh et al., 2018); decreased activation of various mesolimbic and mesocortical areas (van der Vegt et al., 2013; Vriend et al., 2015); and changes in connectivity in the default-mode, central executive, and salience networks (Tessitore et al., 2017b) in PD + ICD, indicating more widespread network-level correlates of predisposition to PD + ICD.

**FIGURE 1:**
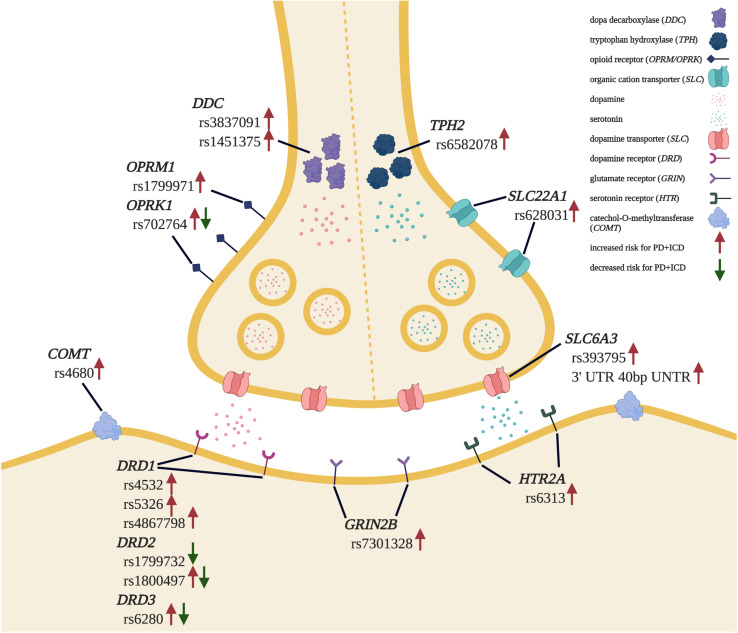
Genetic landscape of PD + ICD risk. Genetic polymorphisms found to be associated with increased and/or decreased risk for ICD development in PD patients are indicated. Adapted from “Synaptic Cleft” by BioRender.com (2020). Retrieved from https://app.biorender.com/biorender-templates.

Finally, genetic association studies have demonstrated that the heritability of PD + ICD is around 57% (Kraemmer et al., 2016). Polymorphisms in genes encoding dopamine receptors (Lee et al., 2009; Zainal Abidin et al., 2015; Krishnamoorthy et al., 2016; Erga et al., 2018; McDonell et al., 2018; Redenšek et al., 2020), dopamine transporters (Cilia et al., 2016; Cormier-Dequaire et al., 2018; Redenšek et al., 2019, 2020), and proteins involved in dopamine metabolism (Ziegler et al., 2014; Kraemmer et al., 2016; Jesús et al., 2020) have been linked to increased PD + ICD risk. Interestingly, genetic modifiers of ICD risk appear to extend beyond the dopamine axis, including genes involved in glutamate (Lee et al., 2009; Zainal Abidin et al., 2015), serotonin, and (Lee et al., 2012; Cilia et al., 2016; Kraemmer et al., 2016) opioid (Kraemmer et al., 2016; Cormier-Dequaire et al., 2018; Erga et al., 2018) signaling, and other signal transduction genes (Hoenicka et al., 2015; Prud’hon et al., 2020) in PD + ICD. These data, summarized in Figure 1, illustrate a complex genetic landscape of ICD risk and implicate a range of non-dopaminergic systems that may be harnessed for treatment.

### Mechanisms Underlying PD + ICD: Rodent Studies

The psychopharmacological profile of D2R agonists in healthy rodents is complex: D2R agonists increase food consumption (Martin-Iverson and Dourish, 1988) and overall wakefulness (Qu et al., 2010) but tend to decrease locomotor activity (Li et al., 2010). The rewarding or aversive properties of DAAs in healthy rodents can be assessed through conditioned place preference/aversion assays (CPP/CPA) wherein injection with a DAA is repeatedly coupled with one chamber, while the control stimulus (saline injection) is paired with another chamber. On test day, the subject can freely explore all chambers. D1R agonists induce significant CPA, while D2R agonists enhance CPP, illustrating the predominant role of D2Rs in the reinforcing properties of DAAs (Zengin-Toktas et al., 2013). While some studies have reported that D2R agonist-mediated reinforcement is similar in both sham and PD-induced rats (Engeln et al., 2013; Zengin-Toktas et al., 2013), other studies have observed that lower doses of DAAs are sufficient to induce CPP in PD-induced rats compared with sham controls (Riddle et al., 2012; Loiodice et al., 2017). Since striatonigral degeneration itself results in a reduction in striatal dopamine levels, the subthreshold effects of DAAs may be related to baseline underactivation of D2Rs (Gallo, 2019). Further studies are clearly required to clarify the upstream mediators and downstream outcomes of D2R agonists in PD-like circuits.

**FIGURE 2:**
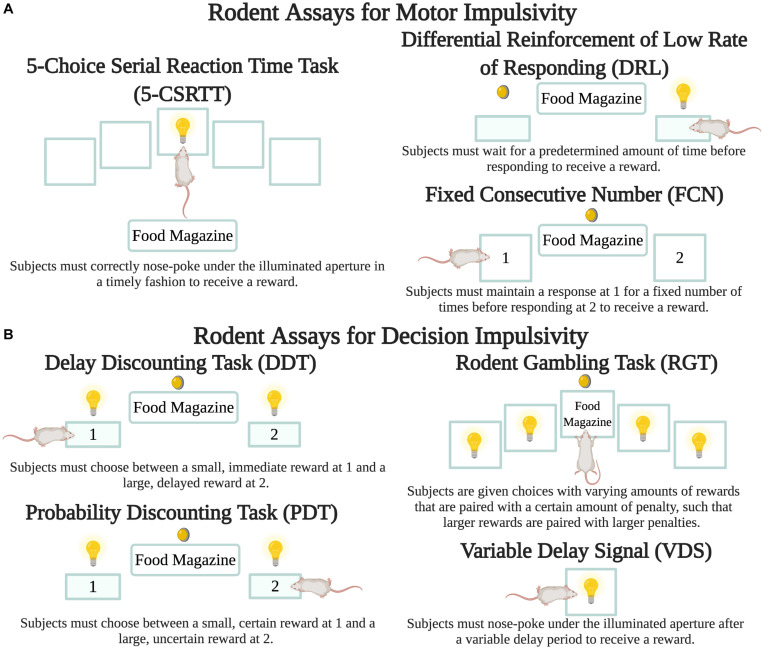
Rodent assays to measure impulsivity. While there are a number of variants for each protocol, the figure depicts the basic details of each. Lightbulbs are cue lights, circular lights are house lights, filled-in rectangles are pressable levers, and transparent squares are apertures for nose pokes. **(A)** Motor impulsivity can be measured through the 5-CSRTT (Higgins and Silenieks, 2017) as well as the DRL and FCN schedules (Engeln et al., 2016). **(B)** Decision impulsivity can be measured through the DDT (Tedford et al., 2015), PDT (Rokosik and Napier, 2012), and RGT (Tremblay et al., 2019). VDS can measure both motor and decision impulsivity (Jiménez-Urbieta et al., 2020). Created with BioRender.com.

A variety of tasks have been developed to measure impulsivity in rodents through quantitative assessments of the speed and accuracy of decision-making (Figure 2). For instance, in the 5-choice serial reaction time task (5-CSRTT) subjects are placed in an operant chamber with five apertures and a food reward tray and trained to receive a reward when they correctly nose-poke under the illuminated aperture in a timely fashion. Here motor impulsivity and compulsivity are reflected in the frequency of premature and perseverative responses, respectively (Higgins and Silenieks, 2017). In one study, PD-induced rats displayed a significant increase in premature response rate immediately following lesioning of the SN pars compacta, and DAA treatment further exacerbated this effect (Jiménez-Urbieta et al., 2019). Protocols that incorporate differential reinforcement of low rate of responding (DRL) and fixed consecutive number (FCN) schedules can also assess rodent motor impulsivity. In DRL, subjects must wait for a predetermined amount of time before responding to get a reward. In FCN, subjects must maintain their response at one station for a fixed number of times before responding at a second station to get a reward. One study incorporating DRL and FCN protocols found that nigrostriatal lesioning was sufficient to cause an increase in both types of motor impulsivity. DAA treatment exacerbated this phenotype, such that following treatment, rats that exhibited the highest rates of impulsivity were lesioned and also displayed high levels of pre-lesion impulsivity (Engeln et al., 2016).

Genetic models of PD have received some emphasis. Mice with a deletion in *SNCA* (encoding alpha-synuclein) displayed decreased motor impulsivity compared with wild-type mice (Peña-Oliver et al., 2012). In a follow-up study, it was determined that while *SNCA* deletion decreases motor impulsivity, it does not affect risky decision-making, measured using the rodent gambling task (RGT), described at the end of this section (Peña-Oliver et al., 2014). The involvement of other neurotransmitter systems in impulsivity has also been revealed through rodent studies. When mice with striatal and PFC lesions performed the 3-CSRTT, a variant of the 5-CSRTT with fewer choices, the amounts of striatal dopamine and norepinephrine were lower and prefrontal serotonin was the same for lesioned mice compared to wild-type mice. However, the ratio of 5-hydroxyindoleacetic acid (a serotonin metabolite) to serotonin was significantly decreased in the PFC of lesioned mice and strongly correlated with increased premature responses, suggesting a mediating role for these neurotransmitters in PD + ICD (Maiti et al., 2016).

In the delay discounting task (DDT) subjects initially prefer the large reinforcer, but as the delay to this reinforcer increases, preference for the smaller and rapid reward grows. This shift in preference is significantly enhanced in treatment-naïve rats with dopaminergic lesions of the dorsolateral striatum (Tedford et al., 2015). However, another DDT study found that lesioning of the dorsal striatum alone did not exacerbate impulsivity (Magnard et al., 2018). The probability discounting (PDT), variable delay-to-signal (VDS) and gambling tasks are also used to assess risky decision-making. In PDT, rodents are presented with choices with two possible outcomes- a small but certain reward or a larger but uncertain reward. As trials progress, the likelihood of receiving the larger reward decreases. Here impulsivity is choosing the larger reward, regardless of whether there is a high or low probability of attaining the reward. Using PDT, pramipexole increased preference for the large reinforcer by approximately 30–45% even at the greatest uncertainty levels in PD-induced rats (Rokosik and Napier, 2012). Even when the expected value of both options remains the same, such as when the uncertain outcome is twice the size as the guaranteed reward but only delivered on 50% of trials, chronic ropinirole administration increased choice for the large-but-uncertain outcome (Tremblay et al., 2017; Russell et al., 2020). The size of the effect was comparable in lesioned and non-lesioned rats.

In the VDS task, a visual stimulus signals the start of a time period during which a nose-poke response is rewarded after a variable delay period (Carvalho et al., 2017). Premature responses during the delay period reflect impulsivity, while perseverative responses after a correct response reflect compulsivity. Using VDS, low pramipexole doses (0.25 mg/kg) increased premature responses (motor impulsivity) and higher pramipexole doses (3 mg/kg) additionally precipitated delay intolerance (decision impulsivity). Moreover, “pre-existing” measures of impulsivity and those measured after lesioning of the dorsolateral striatum were both positively associated with impulsivity following DAA administration (Jiménez-Urbieta et al., 2020). Finally, in the RGT, subjects are generally given choices with varying amounts of rewards that are paired with a certain amount of penalty, such that larger rewards are paired with larger penalties and therefore result in fewer rewards during the course of a session (de Visser et al., 2011). An RGT study showed that chronic ropinirole significantly increased premature responses but did not alter preference for uncertain outcomes when the DAA was given following task acquisition (Tremblay et al., 2019). In the rodent slot machine task (rSMT) subjects respond to three flashing lights that are analogous to the three wheels of a slot machine. If all three lights are set to “on,” the subject needs to respond on the left-hand lever to collect its food reward. On any other trial type, the subject should start a new trial, equivalent to “rolling again,” or incur a time-out penalty. Using rSMT, subjects performed nearly twice the number of trials, and were less sensitive to loss events within the task following chronic ropinirole administration (Cocker et al., 2017, 2019).

### Treatments for PD + ICD

Currently, the most common method to manage PD + ICD is withdrawing DAA treatment following ICRB manifestation. However, this management option has many caveats, including the possibility of dopamine agonist withdrawal syndrome (exacerbation of motor and certain psychiatric symptoms following DAA withdrawal), ICD onset due to other treatments, and behavioral relapse of ICRBs even following DAA elimination (Kelly et al., 2020). A number of pharmacologically distinct psychotropic agents have been examined as interventions, but consensus treatment recommendations are still absent as these studies have typically employed small sample sizes. Positive results have been observed with naltrexone, an opioid antagonist (Bosco et al., 2012; Verholleman et al., 2020); citalopram, a selective serotonin reuptake inhibitor (SSRI) (Ye et al., 2014); atomoxetine, a selective norepinephrine reuptake inhibitor (Kehagia et al., 2014; Rae et al., 2016); valproate, an anticonvulsant mood stabilizer (Hicks et al., 2011); clozapine, an atypical antipsychotic (Rotondo et al., 2010); and amantadine, a weak N-methyl-D-aspartate (NMDA) receptor antagonist (Thomas et al., 2010). However, naltrexone (Papay et al., 2014), various SSRIs (Bosco et al., 2012; Jeon and Bortolato, 2020), amantadine (Thomas et al., 2010; Weintraub et al., 2010b), and several atypical antipsychotics and glutamatergic modulators (Jeon and Bortolato, 2020) have also been reported to have no or negative impact on PD + ICD. In rodent PD models, positive effects have been observed with agonists of G protein-coupled receptor 52 (Russell et al., 2020); propranolol, a β-adrenoceptor antagonist (Cocker et al., 2019); and mirtazapine, an atypical antidepressant (Holtz et al., 2016). Whether non-pharmacological interventions such as deep brain stimulation (DBS) ameliorates or worsens ICRBs in PD patients is still debated. While most human studies indicated that DBS improves PD + ICD, some have reported the development of short-term *de novo* ICDs following DBS (Amami et al., 2015; Rossi et al., 2017; Kim et al., 2018; Lo Buono et al., 2020; Santin et al., 2020). In rodents, decreased risk-taking and loss-chasing was reported following lesioning to the subthalamic nucleus (STN), possibly pinpointing this region as a target for DBS and other treatments for PD + ICD (Breysse et al., 2020). While DBS to the STN reduced risky decision-making in rodents with high levels of pre-existing impulsivity (Adams et al., 2017), in PD-induced rodents, DBS to the STN (Anderson et al., 2020) and globus pallidus internus increased impulsivity (Summerson et al., 2014). As a field, these data point to the need for more comprehensive therapeutics that simultaneously alleviate psychiatric and motor symptoms in patients with PD + ICD.

## Conclusion

Understanding PD + ICD pathophysiology remains a work in progress. While the role of DAAs is generally accepted, the specific molecular interactions that precipitate ICRBs are largely unknown. Human studies have looked into alterations in dopamine receptor subtype expression, dopamine transporter expression, neural networks, and genetic polymorphisms that may predispose PD patients to ICD development, building a complex working model of PD + ICD etiology. Rodent models of PD are essential to test these specific hypotheses, and we are blessed to have access to a rich complement of preclinical protocols (Figure 2) to quantitatively dissect and distinguish features of ICRBs in rodents. These models and assays also provide a substrate to investigate pharmacological and non-pharmacological treatments that ameliorate ICRBs and should be complemented with simultaneous assays of PD motor dysfunction. Not surprisingly, many ICD risk-altering genetic variants (Figure 1) have been linked to other neuropsychiatric syndromes marked by impulsivity and compulsivity, including substance dependence, pathological gambling, and attention-deficit/hyperactivity disorder (Hollander and Rosen, 2000). Thus, cellular and neuroanatomical insights gathered from the study of ICDs in PD patients may have broad relevance beyond PD alone.

## Author Contributions

AA wrote the first draft of the manuscript. All authors contributed to manuscript revision and approved the submitted version.

## Conflict of Interest

The authors declare that the research was conducted in the absence of any commercial or financial relationships that could be construed as a potential conflict of interest.
